# The Census

**Published:** 1921-06-25

**Authors:** 


### The Hospital

The Workers' Newspaper of Administrative Medicine and Institutional Life. Administration. National Insurance and Health.

No. 1829, Vol. LXX. SATURDAY, JUNE 25, 1921. PRICE TWOPENCE

### THE CENSUS.

The numbering of the people took place on Sunday, and the results are certain to be interesting and may be extremely valuable. There has always been a traditional dislike in this country of statistics, and the Census itself in past times has been regarded with aversion as an unwarranted State inquisition into that sacred thing—the English family life. It would be untrue to say that the 1921 Census-taking was a popular event, but the public attitude towards this national method of obtaining statistics has undoubtedly been greatly modified, and probably the vast number of persons who filled in their forms on Sunday realised that they were not performing an idle task for the lucrative pleasure of a Government Department, but that they were actively assisting in a beneficent investigation. Preventive medicine, as many medical correspondents have pointed out, has owed a good deal in the past to the Census, and the debt would have been still greater if the new groupings which were provided in the 1921 Census had been thought of and introduced a generation ago. From the figures to be collected from the new Census it will be possible to make an analysis of industrial mortality, and the Ministry of Health will also be enabled to gain important information of the real housing needs in industrial areas, and of the gaps in local transport. In normal times, too, the unemployment figures would have been of infinite service to the State, but we are afraid their significance will be greatly lessened through the abnormal conditions of the general industrial crisis. In some quarters it has been suggested that the Ministry has lost a priceless opportunity by not combining with the Census a series of questions designed to obtain definite medical information, particularly with the object of the collection of complete statistics as to the national physique. This proposal was, in our opinion, impracticable for one reason and unnecessary for another. In the first place, anyone who has examined the Census forms carefully must entertain a reasonable doubt whether, even with the instructions by which they are accompanied, they are not already so complicated as to endanger the very object for which they have been compiled. The intelligent and patriotic citizen will not have experienced any difficulty in filling them up, but there are large numbers of illiterate, unintelligent, and indolent persons who must either have found them very confusing or involving too much mental labour or requiring information (simple though the demand seems) which they were not able to disclose. The collectors had instructions to render assistance in all cases where it was required, but in some areas of the large cities it must have been a physical impossibility for the collectors to fulfil this task literally. We venture to think there will be a very substantial proportion of incomplete and inaccurate returns. What would have been the case if various details of medical information had also been asked for? Undoubtedly much greater incompleteness, and a mass of inaccuracies that would have rendered such returns valueless, and even dangerous to accept as official records. Moreover, it seems to be forgotten that a much more satisfactory method of obtaining systematic statistical information of this kind already exists in the system of Medical Records established by the National Insurance Commission. A great outcry was raised on the announcement of this new method, but it has now died down, and it is generally recognised that it will in the long run bring great advantage to the State. The records cannot, of course, be complete, but they are at least accurate, and a time may come when it will be possible to develop the system on a real national basis.

